# External Lumbar Drainage to Abort Severe Traumatic IntraCranial Hypertension Phase 1 Randomized Clinical Trial: Scientific Rationale and Methodology: An MTBI^2^ Study

**DOI:** 10.1227/neuprac.0000000000000176

**Published:** 2025-10-20

**Authors:** Halinder S. Mangat, Gregory Hawryluk, Linda M. Gerber, Elizaveta Bokova, Jamshid Ghajar

**Affiliations:** * Brain Trauma Foundation, Palo Alto, California, USA;; ‡ Department of Neurology, Kansas University Medical Center Research Institute, Kansas City, Kansas, USA;; § Department of Neurology, Uniformed Services University of Health Sciences, Bethesda, Maryland, USA;; ‖ Cleveland Clinic Lerner College of Medicine, Case Western Reserve University School of Medicine, Cleveland, Ohio, USA;; ¶ Department of Surgery, Uniformed Services University of Health Sciences, Bethesda, Maryland, USA;; # Department of Population Health Sciences, Weill Cornell Medical College, New York, New York, USA

**Keywords:** Cerebrospinal fluid, External lumbar drainage, Intracranial pressure, Randomized controlled trial, Traumatic brain injury

## Abstract

**BACKGROUND AND OBJECTIVES:**

There has been renewed interest in the use of external lumbar drainage (ELD) of cerebrospinal fluid (CSF) to reduce intracranial pressure after traumatic brain injury (TBI). Although the evidence is retrospective and thus confounded by selection bias, it is critical that a phase 1 trial systematically identify “select” patients who would gain benefit of intracranial pressure (ICP) reduction from lumbar drainage. The aim of this study is to evaluate safety and feasibility of controlled CSF ELD in reducing ICP burden and improving outcomes of select severe TBI patients.

**METHODS:**

The study will include 30 severe TBI patients with a Glasgow Coma Scale score ≤9 who will be randomized to usual treatment, usual treatment plus early ELD placement if initial ICP ≤20 mm Hg, or late ELD if ICP ≥20 mm Hg as first intervention in tier 2 of Seattle International Brain Injury Consensus Consortium protocol.

**EXPECTED OUTCOMES:**

It is expected that the incidence of severe neurological worsening events as per Seattle International Brain Injury Consensus Consortium will not be different between the control and intervention groups.

**DISCUSSION:**

A multitude of clinical trials to advance or discover new treatments of severe TBI have remained unfruitful. Recent understanding of the glymphatic system in the brain is suggestive that extraventricular CSF drainage may be very useful in reducing ICP. The natural avenue for this is through ELD. To avoid past mistakes by jumping into an advanced stage trial, we intend to conduct a phase 1 safety and feasibility trial to identify ‘select’ patients in whom ELD may be performed safely and may serve as a significant change of therapeutic approach to such patients with traumatic intracranial hypertension.

**ABBREVIATIONS:**
cIRB: centralized Institutional Review Board
DSMB: Data and Safety Monitoring Board
ELD: external lumbar drainage
KUMC: Kansas University Medical Center
LAR: legally authorized representative
MTBI: Military Traumatic Brain Injury Initiative
NPI: Neurological Pupillary index
SAEs: serious adverse events
TBI: traumatic brain injury.

## GENERAL INFORMATION

**Sponsor:** Military Traumatic Brain Injury Initiative (MTBI^2^), Bethesda.

**Principal Investigators:** Halinder S. Mangat, MD, MSc, Director of Research, Brain Trauma Foundation, Palo Alto; Kansas University Medical Center Research Institute, Kansas; Gregory Hawryluk, MD, PhD, Medical Director, Brain Trauma Foundation, Palo Alto; Cleveland Clinic Foundation, Cleveland, Ohio; Jamshid Ghajar, MD, PhD, President, Brain Trauma Foundation, Palo Alto.

**Steering committee:** Halinder S. Mangat, MD, MSc; Gregory Hawryluk, MD, PhD, Jamshid Ghajar, MD, PhD., Linda M, Gerber, PhD, Stefan Wolf, MD, PhD.

**Statistician:** Linda M. Gerber, PhD.

**Clinical Trial Research Fellow**: Elizaveta Bokova, MD, University of Kansas Medical Center, Kansas City, KS.

**Clinical Trial Coordination:** Clinical Operations Division, Military Traumatic Brain Injury Initiative (MTBI^2^), Bethesda.

**Primary Clinical Site and Central Institutional Review Board:** University of Kansas Medical Center, Kansas City, KS; Uniformed Services University, Defense Health Agency Institutional Review Board serves as secondary IRB.

**Participating Sites:** University of Kansas Medical Center, Kansas City, Kansas; University of Texas Medical Center, San Antonio; Texas; University of Texas—Southwestern/Parkland Hospital, Dallas, Texas; University of Florida, Gainesville, Florida; Brookes Army Medical Center, San Antonio, Texas; University of Texas, Hermann Memorial Hospital, Houston, Texas.

**Medical Monitor:** Ross Puffer, MD. Uniformed Services University of Medical Sciences, Bethesda.

**DSMB:** Guy Rosenthal, MD, PhD (chairperson), Claudia Robertson, MD, Francis Bernard, MD, Randy Bell, MD.

**Trial Status:** Commenced and enrolling. Anticipated completion date: December 30, 2026.

**Trial Registration:** At clinicaltrials.gov, NCT05889650.

## BACKGROUND AND RATIONALE

Management of moderate-severe traumatic brain injury (TBI) consists of early screening, diagnosis, resuscitation, and surgery (if indicated), followed by specialized neurological intensive care to treat raised intracranial pressure (ICP).^1-3^ Beyond surgery, the cornerstone of TBI treatment pertains to management of elevated ICP which has been associated with improved outcomes by mitigating secondary injury.^4-10^ However, in the past 2 decades, few trials have evolved the management of severe TBI, most failing in phase 3.^11-14^

In an “Opinion” published in JAMA, titled *“Will We Ever Make Headway in Severe Traumatic Brain Injury Treatment Trials?*,*”* the authors emphasize that *“the selection of the right patients for the right therapy is essential if studies are to identify effective treatments.”*^15^ Such a paradigm change may involve *“improving an existing, proven and effective therapeutic intervention in the management of severe TBI and make it more effective.”* One such intervention is cerebrospinal fluid (CSF) drainage to lower ICP.

We previously conducted a systematic review of lumbar CSF drainage in patients with severe TBI that included 9 articles from 6 studies with a total of 110 patients.^16^ Nearly all studies included patients in whom external lumbar drainage (ELD) was performed if raised ICP was refractory to medical therapy. The ICP reduction occurred immediately, and the pooled effect size was −19.5 mm Hg (95% CI: −17.9 to −21.0 mm Hg). Only 1 patient with a previous operated temporal hemorrhage suffered uncal herniation requiring craniectomy but thereafter had a good outcome. Subsequently, more studies have been published demonstrating no increase in the risk of downward cerebral herniation.^17-20^ Indeed, Stevens et al^20^ demonstrated tonsillar elevation rather than descent from ELD, likely due to reduction of supratentorial ICP.

However, we must be vigilant of the selection bias of patients in these retrospective studies. Therefore, we designed a phase 1 safety and feasibility study of early ELD to abort traumatic intracranial hypertension.

## STUDY GOALS AND OBJECTIVES

The objective of this study is to evaluate safety and feasibility of controlled CSF ELD in reducing ICP burden and improving outcomes of select severe TBI patients.

### Specific Aims

#### Primary Aims

To determine whether controlled CSF external lumbar drainage to reduce ICP burden after severe TBI in select patients

- is associated with an increase incidence of neuroworsening events (*Safety*)
- can be successfully performed when indicated (*Feasibility*)

#### Secondary Aims

To determine whether routine quantitative pupillometry can be used for safety determination for ELD by evaluating:

- Correlation of automated pupillometry-derived Neurological Pupillary Index (NPI) with ELD safety score
- If NPI is temporally reduced before occurrence of any “critical neuroworsening event”

## STUDY DESIGN

The aim of this study is to determine the safety and feasibility of ELD to reduce ICP in select severe TBI patients in a phase 1 clinical trial in a multisite randomized, allocation-concealed, open-label, safety and feasibility clinical trial. The study protocol is written in accordance with the ‘Standard Protocol Items: Recommendation for Interventional Trials’ guidelines.

### Human Subjects

#### Inclusion Criteria

- 18 to 65 years of age
- Glasgow Coma Scale (GCS) score 3 to 8
- ICP monitor in place
- Pupils symmetric and bilaterally reactive
- Midline shift ≤ 5 mm at the level of foramen of Monro on admission or postoperative brain CT
- Patent (complete or partial) quadrigeminal cisterns on admission or postoperative brain CT
- Enrollment up to 48 hours after injury (rescreening of patients can be performed if surgery changes exclusion criteria). First randomization and intervention can be commenced within 48 hours of injury
- ELD safety score ≥5

#### Exclusion Criteria

- Cisterns on CT completely effaced
- GCS 3 with dilated and fixed pupils
- Uncal or tonsillar herniation on admission or postoperative brain CT
- Temporal lobe contusions with effaced ipsilateral cistern
- Penetrating TBI
- Primary decompressive craniectomy
- Pregnancy
- Prisoners
- Patients previously lacking capacity to consent or refuse treatment, or with advanced directives to forego aggressive care
- Preexisting conditions affecting functional status or life expectancy to less than 1 year
- Contraindications for ELD placement: uncorrected coagulopathy, unreversed use of anticoagulants or antithrombotics, thrombocytopenia <50 000, uncorrected international normalized ratio (INR) > 1.4, or severe spinal deformity.
- Posterior fossa hemorrhage

## METHODOLOGY

### Usual Treatment

All patients will receive usual treatment as per evidence-based guidelines and Seattle International Brain Injury Consensus Consortium (SIBICC) algorithm (Table 1).^21,22^ Either ventricular or parenchymal ICP monitors may be used. Hourly neuromonitoring will be performed including automated infrared pupillometry, GCS score, ICP, and motor examination. The NPI is an ordinal index ranging from 0 to 5, with a value of less than 3, considered abnormal pupillary reactivity based on prospective studies.^23,24^

**TABLE 1.** SIBICC Treatment Algorithm

Tier 0
Admission to ICU
Endotracheal intubation and mechanical ventilation
Serial evaluations of neurological status and pupillary reactivity
Elevate HOB 30°-45°
Analgesia to manage signs of pain (not ICP directed)
Sedation to prevent agitation, ventilator asynchrony, etc. (not ICP directed)
Temperature management to prevent fever
Measure core temperature
Treat core temperature above 38°C
Consider antiseizure medications for 1 wk only (in the absence of an indication to continue)
Maintain CPP initially ≥60 mm Hg
Maintain Hb >7 g/dL
Avoid hyponatremia
Optimize venous return from head (eg, head midline, ensure cervical collars are not too tight)
Arterial line for continuous blood pressure monitoring
Maintain SpO_2_ ≥94%
Recommended interventions
Insertion of a central line
End-tidal CO_2_ monitoring
Tier 1
Maintain CPP 60-70 mm Hg
Increase analgesia to lower ICP
Increase sedation to lower ICP
Maintain PaCO_2_ at low end of normal (35-38 mm Hg/4.7-5.1 kPa)
Mannitol by intermittent bolus (0.25-1.0 g/kg)
Hypertonic saline by intermittent bolus
CSF drainage if EVD in situ
Consider placement of EVD to drain CSF if parenchymal probe used initially
Consider antiseizure prophylaxis for 1 wk only (unless indication to continue)
Consider EEG monitoring
Tier 2
Mild hypocapnia range 32-35 mm Hg/4.3-4.6 kPa
Neuromuscular paralysis in adequately sedated patients if efficacious
Perform MAP Challenge to assess cerebral autoregulation and guide MAP and CPP goals in individual patients
Should be performed under direct supervision of a physician who can assess response and ensure safety
No other therapeutic adjustments (ie sedation) should be performed during the MAP Challenge
Initiate or titrate a vasopressor or inotrope to increase MAP by 10 mm Hg for not more than 20 minutes
Monitor and record key parameters (MAP, CPP, ICP, and PbtO_2_) before during and after the challenge
Adjust vasopressor/inotrope dose based on study findings
Raise CPP with fluid boluses, vasopressors and/or inotropes to lower ICP when autoregulation is intact
Tier 3
Pentobarbital or thiopentone coma titrated to ICP control if efficacious
Secondary decompressive craniectomy
Mild hypothermia (35-36°C) using active cooling measures

CPP, cerebral perfusion pressure; CSF, cerebrospinal fluid; HOB, head of bed; ICP, intracranial pressure; ICU, intensive care unit; MAP, mean arterial pressure; PbtO2, partial pressure of brain oxygen.

### Intervention

ELD placement will be performed bedside or in operating room if patient is undergoing surgery but not under radiographic guidance and connected to the LimiTorr™ drainage system (Integra Neurosciences), and zero-leveled at the level of the tragus. The LimiTorr™ provides volume-limited drainage and shuts off at a maximum drainage of 10 mL/hour, by always leaving 10 mL of fluid in the 20 mL shut-off-burotrol. The burotrol will be primed with 10 mL of sterile water or saline and will only be drained to a maximum mark of 10 mL on the burotrol—stickers will be placed on the burotrol to ensure adherence to protocol. However, should overdrainage occur in a rare instance, the ELD will be clamped for 1 hour.

### Randomization

The study will enroll 30 severe TBI patients who will undergo 2-stage randomization into usual treatment, usual treatment plus early ELD placement if ICP ≤ 20 mm Hg, or late ELD if ICP ≥ 20 mm Hg (Figure). The process of randomization will be centralized through a secure computerized site.

**FIGURE.**
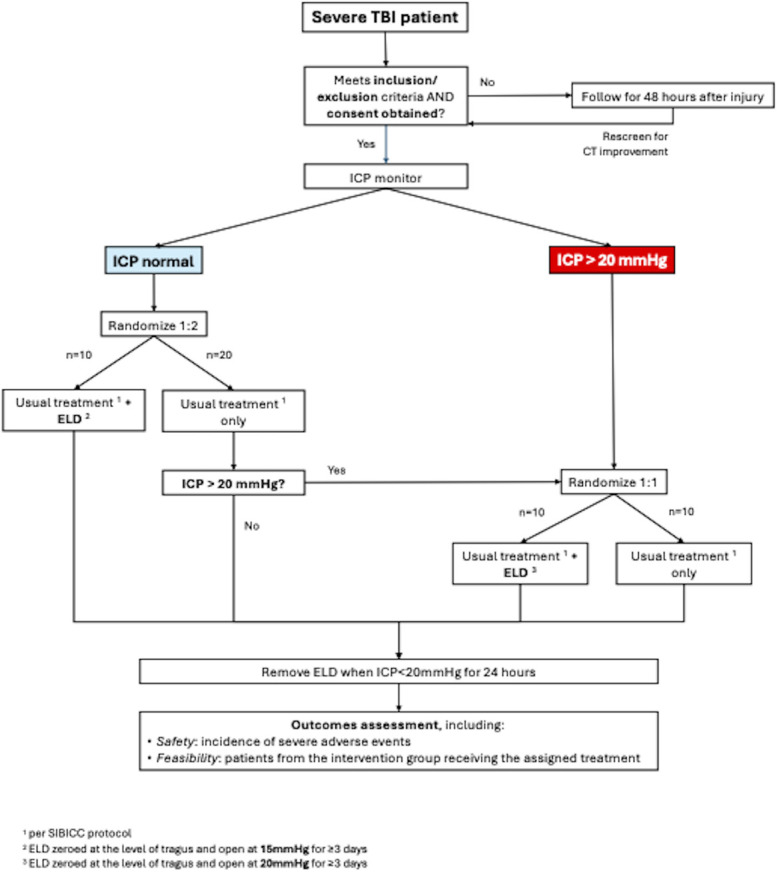
Randomization process. ELD, external lumbar drainage; GCS, Glasgow Coma Scale; ICP, intracranial pressure; SIBICC, Seattle International Severe Traumatic Brain Injury Consensus Conference; TBI, traumatic brain injury.

### Outcomes

#### Primary Outcomes

- Feasibility: We will calculate proportion of patients who are randomized to each intervention and receive it.
- Safety: Frequency of critical neuroworsening adverse events between the intervention and treatment arms as adjudicated by Data and Safety Monitoring Board (DSMB).

#### Secondary Outcomes

Association of absolute NPI and between-eyes NPI difference with.

- ELD safety score on screening CT head,
- Temporal occurrence of neuroworsening events.

## DISCUSSION

Severe traumatic brain injury carries a high morbidity and mortality. No dramatic shift has been made in novel treatments in its management, and mortality rate remains stagnant. Recent literature as reviewed in the introduction seems to be supportive of the use of ELD as a treatment modality for refractory intracranial hypertension. However, the data are skewed significantly by selection bias of patients. Patients in all the studies were refractory to medical therapy and were selected based on different criteria in different studies, and thus, it is not possible to either make a generalized inclusion criteria nor is it possible to ascertain what proportion the included patients constitute of the whole cohorts with similar findings.

Therefore, we believe that instead of moving into a very tempting phase 3 study, that ELD use should be systematically investigated by describing unifying but slightly conservative inclusion/exclusion criteria in this study, and the investigation at the foundation level with a phase 1 trial, the results of which will inform possibly modifying the protocol during the phase 1 trial, or to construct a meaningful protocol for a phase 2/3 trial after ELASTIC.

The additional benefits of the ELASTIC trial are that non-neurosurgical medical professionals could be trained to do the procedure in austere environments. Serial lumbar punctures may be an alternative approach to patient management in austere environments. Given 90% of TBI occurs in austere environments, there are several such environments even in the United States as in Alaska, Hawaii, the Dakotas and other rural areas. Other such circumstances arise in low- and middle-income countries, in combat, refugee camps, and natural disasters.

## TRIAL STATUS

The ELASTIC trial has begun enrolling at 4 sites: University of Kansas Medical Center, Kansas City; University of Florida, Gainesville; University of Texas Health Science Center at San Antonio; and University of Texas—Southwestern, Dallas. Three further sites are expected to be added in the next 2 months.

## SAFETY CONSIDERATIONS

### Risks to Human Subjects

Critical neuroworsening events related to ELD and CSF drainage are neurological worsening events such as decrease in GCS score, development of new motor deficit, or a cerebral herniation event, and rarely death (Table 2). Given the greatest risk of neuroworsening from herniation is soon after drainage is commenced, all patients will have neurological checks ie GCS score, ICP measurement, automated pupillometry for pupillary size, symmetry and NPI performed every 15 minutes for 1 hour after ELD placement, and start of CSF drainage. If NPI drops <3 within the first hour, a head CT may be obtained to review any craniocaudal shift. After the first hour, measurements will be performed hourly.

**TABLE 2.** Neuroworsening Events

Critical neuroworsening events per SIBICC protocol
1. Spontaneous decrease in the GCS motor score of ≥1 points (compared to previous exam)
2. New decrease in pupillary activity
3. New pupillary asymmetry or bilateral mydriasis
4. New focal motor deficit
5. Herniation syndrome or Cushing Triad which requires an immediate physician response

GCS, Glasgow Coma Scale.

Based on inclusion/exclusion criteria, we analyzed the severe TBI BTF-New York State TBI-trac database from 1997 to 2007, with 1100 patients. The inclusion criteria applied to 47% of all severe TBI patients admitted over 10 years to 11 level 1 trauma centers in the state; 22% (95% CI: 19.9%-24.3%) suffered new pupillary asymmetry at any point during admission, 18% (95% CI: 12.6%-25.0%) suffered new motor deficit, and 22.4% (95% CI: 20.2-24.8) experienced death at 2 weeks.

### Mitigation Against Risk of Cerebral Herniation

#### Eligibility

From previous studies, we will use a conservative midline shift of 5 mm, as well as the ELD safety score, ELD will only be performed if safety score is ≥5 (Table 3).

**TABLE 3.** ELD Safety Score^25^

Prepontine cistern	Quadrigeminal cistern	Uncal herniation	Foraminal herniation
Normal: 2	Normal: 2	Absent: 2	Absent: 2
Compressed: 1	Compressed: 1	One side: 1	
Not discernible: 0	Not discernible: 0	Both sides: 0	Present: 0

Total score ≥ 5: LP or LD feasible; ≤ 5: LP or LD not feasible.

#### ELD Drainage Pressure

ELD in patients without high ICP will be set at 15 mm Hg and those with high ICP at 20 mm Hg, with a maximum of 10 mL/h drainage of CSF.

#### ELD Drainage Volume

No more than 10 mL CSF will be drained within 1 hour in either intervention arms; this will be ensured by using the LimiTorr ELD system as described before.

#### STOP Criteria

ELD shall be discontinued if.

1. Any patient has an acute herniation event within 1 hour of ELD placement and CSF drainage
2. There is a decrease of NPI <3 (the abnormal threshold) within 1 hour of ELD placement and CSF drainage that is not reversed by additional tier 1 therapies.
3. The treating team deems continuation of ELD as a clinical risk to patient based on neuroimaging or clinical criteria.
4. If legally authorized representative (LAR) withdraws consent for continuing study

### Adverse Events Reporting

#### Local Institutional and Central IRB

All critical neuroworsening events in intervention arms will be reported to the medical monitor who will make determination of expectedness, and if deemed unexpected will be further reported to local site institutional IRB, central IRB, and DSMB within 24 hours.

#### Data Safety and Monitoring Board

A DSMB of 4 experienced trauma neurosurgeons and neurointensivists of international standing will be constituted, who will review every suspected unexpected critical neuroworsening event, as defined above, in each arm of the study. All critical neuroworsening events will be reported to the medical monitor (a neurosurgeon) within 24 hours, who will review expectedness, and refer all unexpected serious adverse events (SAEs) to the DSMB. If an unexpected SAE is suspected, ongoing ELD CSF drainage will be paused. The DSMB will review all intervention-related critical neuroworsening events and present a decision to the steering committee within 48 hours. A minimum of 3 DSMB members will independently review SAE. If the DSMB concludes that a critical neuroworsening event was not related to intervention, participant may continue study procedures per protocol. If, however, DSMB deems a neuroworsening event to be related to intervention, and if a discrete contributing factor is identified, the steering committee will meet within 7 days to review the report and discuss necessary changes to protocol before further enrollment.

## Follow-up

Follow-up for the study will be till 14 days since injury to determine 2-week mortality. Other outcomes will be measured during intensive care unit stay only.

## DATA MANAGEMENT AND STATISTICAL ANALYSIS

All data including screening, inclusion/exclusion criteria, e-consent, randomization, and clinical data from all patients will be collected directly into a centralized electronic case report form supported by the MTBI^2^ Collection, Access, Sharing, and Analytics (CASA) platform system based in Henry Jackson Foundation Bethesda. National Institute of Neurological Disease and Stroke TBI-related common data elements from the following domains will be used: patient characteristics and history, disease/injury-related events, assessments and examinations, treatment/intervention data, protocol experience, and outcomes and end points (32) (Table 4).

**TABLE 4.** Common Data Elements Collected: Domains, Measures, and Instruments

Domain	Subdomain	Measures/instruments
Participant characteristics and history	Demographics	Age, race/ethnicity, sex/gender, socioeconomic status, military service
	General health history	Significant medical history
Disease/injury Related events	Classification	Baseline risk assessment
		Injury severity score
		Head AIS
	History of disease/injury event	Injury presentation^a^
		Type, place, cause and mechanism of injury
	Discharge information	Discharge destination, time, and vital status
	Second insults	Aspiration, cardiac arrest, hyperventilation, hypotensive episode, seizure
		EEG monitoring
Assessments and examinations	Physical/neurological examination	Glasgow coma scale
		Pupil size, shape, and reactivity
		neurological pupil index
	Vital signs and other body measurements	Height and weight
		ICP monitoring
	Imaging	Cranial CT data
	Laboratory tests	Laboratory results (including INR and platelet count)
Treatment/intervention data	Surgeries and other procedures	ER/admission therapeutic procedures
		Surgical and therapeutic procedures
		Intraoperative management
	Therapies	Therapy intensity level
	Drugs	Prior and concomitant medications
Protocol experience	Participant/subject identification, eligibility, and enrollment	Informed consent
	Off treatment/off study	Study discontinuation/completion
Outcomes and end points	Disability	Glasgow outcome scale
	Adverse events	Severity: common terminology criteria for adverse events v6.0
		Expectedness: NIA adverse event and serious adverse event guidelines
		Causality: NIA adverse event and serious adverse event guidelines
		Neuroworsening events

AIS, Acute Injury Severity; CT, computed tomography; ICP, intracranial pressure; INR, international normalized ratio.

a Date and time of injury, symptom onset, arrival to the hospital, emergency service response, first treatment, admission.

The secure database can only be accessed by personnel certified by the Informatics Division of MTBI^2^ using a secure access card and key. No data can be removed, only data pertaining to a subject being included in trial at any site can be viewed by that site's research personnel. Research team will not have access to the data until data lock is lifted. All patients will be assigned a Global Unique Identifier, and at the end of the trial, all data will be uploaded to the central Federal Interagency Traumatic Brain Injury Repository.

## OUTCOMES

### Primary Outcomes

Both “intention-to-treat” and “treated as per protocol” analyses will be performed. Incidence of critical neuroworsening events will be compared between each arm, and between both ELD groups and control arm. Univariate and multivariable analyses will be performed using baseline variables, hourly NPI change, and volume of CSF drained per day for outcomes, ie frequency of neuroworsening events and death. Multivariable analyses will use stated adverse outcomes as dependent variables, and age group, sex, baseline ELD safety score, and pupillary abnormality as independent variables. Logistic regression will be performed using those variables that have an association with outcome with a *P*-value of ≤.10.

For feasibility, a comparison between proportion of patients in each arm who underwent ELD as per protocol will be performed. Comparison between the 2 ELD groups will be performed. Interactions will be tested for in all analyses.

### Secondary Outcomes

Multivariable analysis will be performed using relevant clinical variables including most recent NPI before neuroworsening event, hourly change in NPI as additional independent variables introduced iteratively with neuroworsening outcomes as dependent variable.

## QUALITY ASSURANCE AND PROJECT MANAGEMENT

The trial operations are being performed by the Clinical Operations Division at MTBI,^2^, Bethesda. The group is composed of the Director of Clinical Operations, an operations manager, a trials manager, a regulatory operation personal, a safety and protocol adherence reviewer, and 2 information technology personnel. The PI and research fellow complete all activities in accordance with federal requirements. Site training and visits are undertaken by and under the supervision of the Clinical Operations Division staff, and biweekly meetings are held to review screening and screen failures. The steering committee meets every 2 months or more often as needed. Annual reports are submitted to the sponsor.

## EXPECTED OUTCOMES OF THE STUDY

The trial will provide an estimate of safety of ELD use at 2 time points in the chain of TBI care, viz early: before onset of intracranial hypertension and late: after onset of intracranial hypertension. It is hypothesized that the use of ELD in both instances will not increase incidence of SAEs in patients. If this is so, a composite phase 2/3 randomized controlled trial will be designed to test efficacy and effectiveness of the intervention. This will likely be an international multisite clinical trial.

## DURATION OF THE PROJECT

The initial timeline of the study was 2 years; however, due to logistical issues, start of enrollment was delayed by 12 months. Therefore, we plan to extend trial duration by 18 months to complete enrollment of 30 patients. Furthermore, we did not anticipate the high rates of practice of primary decompressive craniectomy in severe TBI patients (close to 40%) which has slowed enrollment.

## ETHICS

The ELASTIC trial has a centralized Institutional Review Board (cIRB) at Kansas University Medical Center (KUMC), Kansas City, KS. All sites perform reliance agreements with KUMC cIRB. All informed consent forms need to comply with local and KUMC IRB requirements and are available in English and Spanish. The Spanish translation is performed by a professional translation service with a certificate of authenticity provided along with. Any protocol amendments are reviewed by cIRB as minor or major, the latter requires secondary IRB approval before implementation across sites.

The study requires a full informed consent from a LAR who can consent in-person or remotely (e-consent) via the CASA platform. All information is entered in the electronic case report form and is tagged to the Global Unique Identifier, the key for which remains locked at each site with site PI access only. LARs may refuse or withdraw consent at any time.
